# Bleeding recurrence in patients with gastrointestinal vascular malformation after thalidomide

**DOI:** 10.1097/MD.0000000000004606

**Published:** 2016-08-19

**Authors:** Haiying Chen, Sengwang Fu, Nan Feng, Huimin Chen, Yunjie Gao, Yunjia Zhao, Hanbing Xue, Yao Zhang, Xiaobo Li, Jun Dai, Jingyuan Fang, Zhizheng Ge

**Affiliations:** Division of Gastroenterology and Hepatology, Shanghai Institute of Digestive Disease, Key Laboratory of Gastroenterology and Hepatology, Ministry of Health, Renji Hospital, School of Medicine, Shanghai Jiao-Tong University, Shanghai, China.

**Keywords:** adverse effects, gastrointestinal vascular malformation, recurrence rate, response rate, thalidomide

## Abstract

Thalidomide may be used for the treatment of gastrointestinal vascular malformation (GIVM), but the long-term response and adverse effects are unknown. Aim to study the recurrence rate of GIVM bleeding after thalidomide treatment, the response to treatment, and the adverse effects.

This was a retrospective study of 80 patients with GIVM treated with thalidomide between November 2003 and November 2013. Patients received a course of 100 mg/day of thalidomide for 4 months and were followed up for at least 1 year. The response rate during follow-up, the recurrence rate after the 1st course of treatment, and the rate of retreatment were assessed. Comorbidities, the need for blood transfusion, yearly bleeding episodes, hemoglobin levels, hospitalization after thalidomide treatment, and the rate of adverse effects were also examined.

The overall response rate during follow-up was 79.5% (62/78). The recurrence rate was 21.0% after the 1st course of thalidomide. The response rate of retreatment was 100%. After thalidomide treatment, yearly blood transfusion amounts, yearly bleeding episodes, and yearly hospitalization numbers were significantly decreased, while hemoglobin levels were significantly increased (*P* < 0.001). Adverse effects were observed in 60.0% (48/80) of the patients. Serious adverse effects were reported in 31.3% (25/80). The overall response rate was 76.7% (23/30) in 30 patients with comorbidities, while the rate was 78.0% (39/50) in patients without comorbidities (*P* = 0.55). The rate of serious adverse effects was similar between the comorbidities (33.3%) and no-comorbidities groups (30.0%) (*P* = 0.76).

Thalidomide showed a good response rate and low adverse effect rate in patients with recurrent gastrointestinal bleeding due to GIVM.

## 1 Introduction

Gastrointestinal (GI) vascular malformation (GIVM) is a hemorrhagic disease involving the entire GI tract. About 90% of cases are located in the small intestine. GIVM is the main cause of occult GI bleeding, particularly in the elderly.^[1]^ Approximately 20% of the patients present iron-deficiency anemia and/or stools with intermittently positive fecal occult blood test. Massive bleeding occurs in about 12% to 27% of the patients^[2,3]^ and 50% of the patients need frequent hospitalization and repeated blood transfusions.^[4]^

Conventional interventional therapies are often ineffective in preventing recurrent GI bleeding and may occasionally be associated with severe complications.^[5–9]^ Some drugs were previously considered promising, but a recent randomized study found that such a treatment was ineffective.^[3]^ Other medicines like danazol,^[10]^ tranexamic acid,^[11]^ desmopressin,^[12]^ and recombinant factor VIIa^[13]^ were considered effective but without confirmation using a randomized controlled study. Junquera et al^[14]^ demonstrated a lower recurrence rate in patients receiving octreotide compared with the placebo group.

An effective and relatively safe pharmaceutical agent is still needed. Thalidomide possesses antiangiogenesis properties via the suppression of the vascular endothelial growth factor,^[15]^ which has been shown to be strongly expressed in GIVM.^[16]^ Thalidomide also possesses immunomodulating, antitumor, and antiinflammatory properties, but its nefarious teratogenic effects may limit its use.^[15,17,18]^ Thalidomide was successfully used for the treatment of recurrent gastric bleeding in a patient with hereditary hemorrhagic telangiectasia (HHT)^[19]^ and for the treatment of cirrhotic patients with severe anemia secondary to vascular malformation.^[20]^

Our group reported a response rate of 71.4% for thalidomide in patients with refractory GI bleeding due to GIVM at 1 year follow-up.^[4]^ Therefore, this study aimed to assess the long-term response of GIVM to thalidomide, as well as the adverse effects.

## 2 Methods

### 2.1 Patients

This was a retrospective study of patients diagnosed with GIVM at the Department of Gastroenterology, Renji Hospital, from November 2003 to November 2013. Because of the lack of a universally accepted classification system for GIVM, radiological diagnoses of angiodysplasia, vascular ectasia, arteriovenous malformation, vascular malformation, or angioma were included in this study.^[21]^

Inclusion criteria were: aged ≥18 years; male or female patients, but female patients were postmenopausal, with posttubal ligation, or females with childbearing potential using any form of birth control; patients had recurrent (≥4 bleeding episodes in the year prior treatment) or refractory bleeding due to GIVM; and all confirmed GIVM without obvious infectious, cancers, or other specific lesions, based on comprehensive examinations.

Refractory bleeding was defined as bleeding that is either unresponsive to conventional therapies (i.e., recurrent bleeding after at least 3 sessions of esophagogastroduodenoscopy or colonoscopy, or at least 1 session of double-balloon endoscope therapy or primary medical approaches) or unsuitable for conventional therapies (i.e., multiple lesions, lesions inaccessible by endoscopic therapy or surgery, or patients unwilling or unable to undergo surgery due to poor health condition).

Angiodysplasia at endoscopy was characterized by focal or diffused venous/capillary lesions presenting as bright red ectatic vessels or pulsatile red protrusions, with surrounding venous dilation or patchy erythema with or without oozing, and endoscopic appearance of GAVE was indicated by longitudinal antral folds converging on the pylorus, containing visible columns of tortuous red ecstatic vessels.^[22,23]^

Exclusion criteria were: cirrhotic or portal hypertension gastropathy; severe cardiac, pulmonary, renal, liver, pancreas, hematological, or rheumatologic comorbidities, uncontrolled diabetes mellitus or hypertension, or renal insufficiency without hemodialysis or peritoneal dialysis; severe bilateral peripheral neuropathy or seizure, thromboembolic disease, or known thalidomide allergy; treatment with any systemic or oral topical corticosteroids, nonsteroidal antiinflammatory drugs, any putative immunomodulators, or antiangiogenic agents; pregnant or lactating; alcohol and/or drug abuse; or poor compliance.

This study was approved by the ethical committee of the Renji Hospital. The need for individual consent was waived by the committee because of the retrospective nature of the study.

### 2.2 Thalidomide treatment and management of adverse effects

In our center, patients received thalidomide only when they had 4 or more bleeding episodes a year as defined by a positive immunoassay fecal occult blood test (iFOBT). Patients received 25 mg thalidomide (Changzhou Pharmaceutical Co., Ltd., Changzhou, China) orally 4 times/day for 4 months. During the treatment period, patients were hospitalized and closely monitored for the 1st week or longer, if required. In patients with recurrent bleeding, another course of thalidomide could be prescribed, but the time between courses had to be at least 1 year.

In the case of an adverse effect, the study medication was temporarily interrupted or permanently discontinued based on the seriousness of the effect or tolerability of the patient. During treatment and follow-up, concomitant therapies such as blood transfusions, iron supplementation, use of hemostatic drugs, and any other symptomatic treatments were performed as necessary. If massive bleeding occurred in the context of poor clinical effect of symptomatic treatment, endoscopic treatment, DSA embolism, or surgery were considered.

Packed red cell transfusion was indicated and recorded when the hemoglobin levels fell below 7.0 g/dL (2 U were administered for 6.1 g/dL ≤ hemoglobin ≤ 7.0 g/dL, 3 U for 5.1 g/dL ≤ hemoglobin ≤ 6.0 g/dL, and 4 U for hemoglobin < 5.0 g/dL).^[24]^ Iron supplementation was allowed when the hemoglobin levels fell between 7.1 and 11.0 g/dL. All patients were followed up until the last patient had been followed up for 1 year after thalidomide treatment.

### 2.3 Data collection

Two qualified research nurses collected demographics, medical and family histories, and all clinical data including any adverse effects during follow-up. All physical examinations, treatments, and follow-up were performed by the same 2 qualified doctors. During follow-up, as a standard follow-up procedure, patients had to report the characteristics of their feces (i.e., color, volume, frequency of defecation, and softness) daily in a diary. All patients were requested to visit the hospital monthly during follow-up and biweekly during treatment.

At each scheduled visit, a physical examination, complete blood counts, and routine iFOBT (using monoclonal colloidal gold color technology) were performed. In case of positive iFOBT, with or without any signs of overt bleeding, iFOBT was performed daily until there were 3 consecutive days with a negative test, in order to ensure whether or not bleeding truly occurred, whether or not bleeding was stopped, and how long it lasted. In addition, during the whole study period, iFOBT was performed daily in the same way in patients with any signs of overt bleeding. Serum chemical laboratory tests, blood coagulation tests, and hepatic and renal tests were performed before and after the treatment. Neuropathy and other adverse effects were assessed monthly. A urine pregnancy test for the β-subunit of human chorionic gonadotropin was performed for female patients with childbearing potential before treatment, biweekly during treatment, and 1 month after treatment.

### 2.4 Treatment response and adverse effects

The patients were divided into 4 groups according to their clinical response after thalidomide treatment: bleeding cessation, bleeding diminution, bleeding diminution when taking thalidomide, and ineffective. Cessation of bleeding was defined as GI bleeding being completely stopped during follow-up. Patients whose yearly bleeding episodes decreased by ≥50% during follow-up were allocated to the bleeding diminution group. Patients whose bleeding stopped when taking thalidomide but relapsed again during follow-up were allocated to bleeding diminution when taking thalidomide group. Patients whose yearly bleeding episodes decreased by <50% during follow-up were attributed to ineffective group.

Short-term recurrence was defined as GI bleeding occurring immediately after drug discontinuation. Long-term recurrence was defined as GIVM recurrence >1 year after thalidomide discontinuance.

The overall response rate was defined as the proportion of patients in the bleeding cessation, bleeding diminution, and bleeding diminution when taking thalidomide groups.

A bleeding outcome was defined as a positive iFOBT. A bleeding episode was defined as being stopped when there were 3 consecutive days with a negative iFOBT test. All bleeding episodes described in this study were attributed to GIVM.

Adverse effects included any unfavorable changes in health including occurrence or worsening of any clinical symptoms, signs, or abnormal laboratory findings during follow-up. The adverse effects were classified as mild (constipation, somnolence, edema, dizziness, headache, pruritus, and dry mouth) or serious (limb numbness, leukopenia, and rash).

### 2.5 Statistical analysis

Continuous variables are reported as means ± standard deviation or as median (range), and were analyzed using 2-sample paired *t* test or Wilcoxon signed-rank test, as appropriate. Categorical variables are presented as frequencies and were analyzed using the Chi-square test or the Fisher exact test, as appropriate. Statistical analysis was performed by a biostatistician blinded to the treatment assignment using SPSS 16.0 (IBM, Armonk, NY). Two-sided *P-*values <0.05 were considered statistically significant.

## 3 Results

### 3.1 Characteristics of the patients

A total of 87 patients diagnosed with GIVM were eligible. Two patients who had severe concomitant diseases were excluded. Three patients refused additional treatments because of drowsiness, constipation, and slight leukopenia. Two patients did not complete the 1-year follow-up. Thus, 80 patients were included. The enrolled subjects in our present study also included thalidomide group patients in our previous study.

Median age was 63.5 (range, 40–87.0) years and most (92.5%, 74/80) were >50 years old. There were 57 (71.3%) women and 23 (28.7%) men. Seventy (87.5%) patients were complaining of overt bleeding including hematemesis (n = 1), hematochezia (n = 15), and melena (n = 54); 10 patients presented occult bleeding only. The median course of GI bleeding was 22 (range, 1–360) months. During the year prior to treatment, the median number of bleeding episodes was 6 (range, 4-48), the median volume of blood transfusion was 750 (range, 0–15,600) mL, and the median hemoglobin level was 64 (range, 17–113) g/L. Median follow-up was 42.6 (range, 12–120) months.

### 3.2 Response to treatment

During the 1st-year of follow-up, the overall response rate was 77.5% (62 of 80), and the rate of bleeding cessation was 41.3% (33/80). Among the patients with bleeding cessation, 3 had a recurrence after the 1st-year (range, 12–14 months) with 4 to 12 bleeding episodes each year. These 3 patients received a 2nd course of thalidomide. After retreatment, their bleeding episodes decreased significantly.

Among patients with decreased bleeding (bleeding episodes decreased by ≥50% during the 1st-year), 3 patients with increased number of bleeding episodes 12 to 26 months after discontinuation also received a 2nd course of thalidomide and achieved satisfactory effect.

Among patients with decreased bleeding during treatment, 5 patients received a median of 2 courses (range: 2–4) of thalidomide. In the ineffective group, 2 patients had to undergo intestinal resection 12 and 15 months after drug discontinuation. One patient had no recurrence over his 7-year follow-up, while the other had 3 bleeding episodes 3 years after surgery.

The overall response rate of the 78 patients during follow-up was 79.5% (62/78) (Table 1). The recurrence rate of GIVM bleeding after the 1st course of thalidomide was 20.7% (13/62). Among 13 patients with recurrent GI bleeding, 11 patients received thalidomide retreatment with an effective rate of 100%.

**Table 1:**
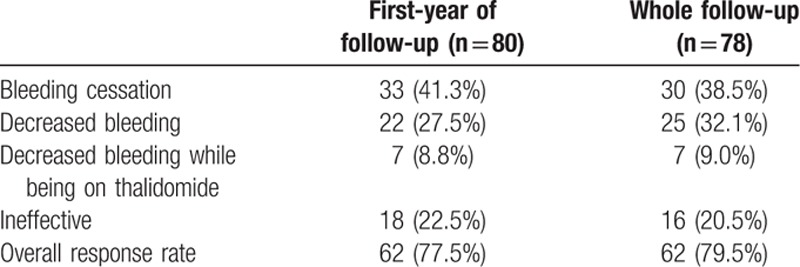
Response to thalidomide.

After thalidomide treatment, yearly bleeding episodes decreased from 8.0 ± 6.4 to 2.3 ± 2.5 (*P* < 0.001). Yearly blood transfusions deceased from 1386 ± 2166 to 315 ± 645 mL (*P* < 0.001). The mean hemoglobin levels were increased from 64 ± 20 to 102 ± 24 g/L (*P* < 0.001). The yearly number of hospitalization for bleeding decreased from 3.0 ± 1.8 to 0.8 ± 1.3 (*P* < 0.001) (Table 2).

**Table 2:**
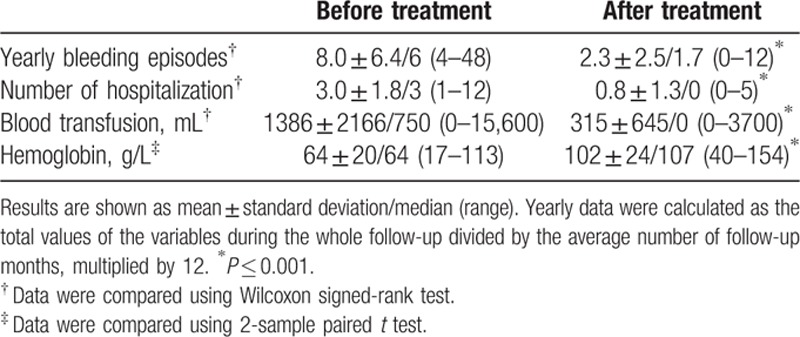
Change of clinical indexes in 80 patients during the entire follow-up.

### 3.3 Comorbidities

Thirty (37.5%) patients had 1 or several preexisting comorbidities. Three patients had cardiac valve disease. Two of these patients were receiving anticoagulation therapy (warfarin) after cardiac valve replacement, which did not affect the response to thalidomide. The 3rd patient was suffering from aortic insufficiency and did not show GI bleeding recurrence during the 70-month follow-up. Only 1 patient had constipation.

Three patients were receiving hemodialysis because of renal insufficiency: 2 showed complete bleeding cessation during follow-up and 1 had a recurrence after 14 months. This patient received a 2nd course of thalidomide, with success. Among these patients, 2 complained of numb limbs.

In 1 patient with hereditary HHT, DSA showed multiple aneurysms in visceral vessels including 2 splenic artery aneurysms, 2 hepatic artery aneurysms, and 1 superior mesenteric artery aneurysm. Besides, this patient had bilateral nasal mucosa telangiectasia and gastric malformation. After 1 course of thalidomide, her bleeding episodes decreased from 12 to 7 times/year, but with leukopenia.

The overall response rate was 76.7% (23/30) among patients with comorbidities, while the response rate was 78.0% (39/50) among those without comorbidities, without difference between the 2 groups (*P* = 0.55) (Table 3).

**Table 3:**
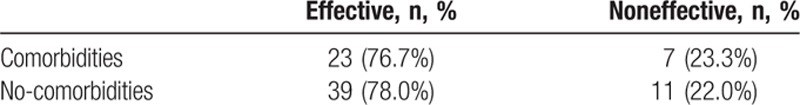
Relationship between comorbidities and clinical efficacy of thalidomide.

### 3.4 Adverse effects

Adverse effects were observed in 60.0% (48/80) of the patients. Serious adverse effects were reported in 31.3% (25/80) of the patients. No adverse effect threatened the life of the patients and disappeared or were relieved after discontinuation of thalidomide or symptomatic treatment.

Of the 30 patients with comorbidities, 22 (73.3%) experienced adverse effects including 10 (33.3%) with serious adverse effects. Of the 50 patients without comorbidities, the rate of overall adverse effects and serious adverse effects were 52.0% (*P* = 0.098) and 30.0% (*P* = 0.76), respectively. There were no differences between the 2 groups (Table 4).

**Table 4:**
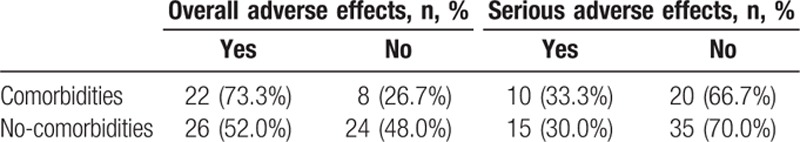
Relationship between comorbidities and adverse effects.

Among the 11 patients with multiple courses of thalidomide treatment, the overall rate of adverse effects and serious adverse effects were 100% (11/11) and 63.6% (7/11), respectively. For patients with a single course of thalidomide, these rates were 53.6% (37/69) (*P* = 0.002) and 26.1% (18/69) (*P* = 0.03), respectively (Table 5).

**Table 5:**
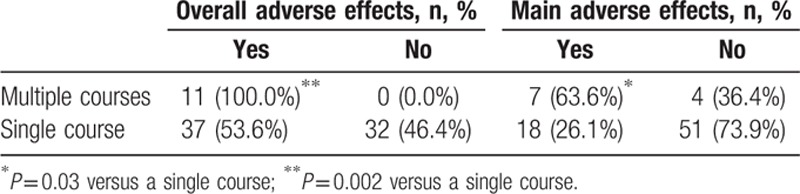
Relationship between thalidomide treatment courses and adverse effects.

## 4 Discussion

Thalidomide may be used for the treatment of GIVM, but the long-term response and adverse effects are unknown. This study aimed to examine the recurrence rate of GIVM bleeding after thalidomide treatment, the response to treatment, and the adverse effects. Results showed that the overall response rate during follow-up was 79.5%. The recurrence rate was 20.7% after the 1st course of thalidomide. The response rate of retreatment was 100%. The overall response rate and adverse effect rate were similar between patients with or without comorbidities.

The efficacy of thalidomide in treating recurrent GI bleeding due to GIVM was previously reported in a number case reports and small case series.^[25–27]^ In this study, the overall effective rates during the 1st year and during the entire follow-up were 77.5% and 79.5%, respectively, similar to our previous randomized controlled study in which the effective rate of thalidomide treatment for GIVM patients was 71.4% during the 1st-year follow-up.^[4]^

The efficacy and safety of thalidomide for GIVM with comorbidities was only discussed in some case series. Draper et al^[28]^ prescribed thalidomide to 7 GIVM bleeding patients with left ventricular assisting device implantation. GI bleeding was stopped in 5 patients and significantly reduced in 2 patients. In this study, the overall effective rate and the rate of main adverse effects of thalidomide in the comorbidities group were 76.7% and 33.3%, respectively, which were similar to the rates of 78.0% and 30.0% in patients without comorbidities, but the strict selection criteria in this study may have introduced a selection bias.

Alam et al^[29]^ reported that thalidomide had a good clinical effect on recurrent GI bleeding from gastric and duodenal angiodysplasias because of hereditary HHT. In this study, 1 patient with gastric body malformation due to HHT failed to respond to thalidomide after 4 months, although her yearly bleeding episodes decreased. This difference might be due to the longer treatment course (16 months) in the study by Alam et al.^[29]^

Previous studies used thalidomide doses of 50 to 400 mg/day. Considering the older age of the patients (average age, 64.6 years) in this study, a low dose was selected (100 mg daily). In this study, the recurrence rate of GIVM bleeding after the 1st course of thalidomide treatment was 20.7% (13/62). Among these 13 patients with recurrence, 11 patients received an average 2 (range: 2–4) course of thalidomide. All patients had decreased bleeding episodes when taking thalidomide or after another course of medication. The effective rate of thalidomide retreatment was 100%. Meanwhile, the rate of serious adverse effects was 63.6% (7/11), which was higher than the percentage of serious adverse effects after a single course of thalidomide (26.1%, 18/69).

Thalidomide carries the risk of adverse effects such as fatigue, constipation, dizziness, peripheral neuropathy, and edema.^[30]^ In this study, the overall rate of adverse effects was 60.0%. All adverse effects were mild-to-moderate and were relieved or disappeared after discontinuation of thalidomide treatment or symptomatic treatment. A new thalidomide analogue (lenalidomide), which is reported to be a more potent immunoregulator with possibly fewer side effects,^[31,32]^ could be used for the prevention of GI bleeding due to GIVM, but studies are required in this specific population of patients.

In addition to angiogenesis, VEGF is also associated with neural regeneration. Therefore, neurotoxicity is an important adverse effect for antiangiogenic drugs.^[33]^ A study demonstrated that suppressing VEGF level by 25% could lead to motor neuron degeneration.^[34]^ Our previous studies found that thalidomide could inhibit VEGF expression in vivo and ex vivo.^[4,35]^ Cavaletti et al^[36]^ confirmed that sensory neurotoxicity of thalidomide was found to be dependent upon the cumulative dose but occurred only when the total dose was relatively high (>20 g); the risk of developing sensory neuropathy was around 10% below this threshold (20 g) but increases with higher doses.^[36]^ In this study, the total dose of a single course of thalidomide was 12 g with a rate of 14.5% of limb numbness, while it increased to 63.6% for 2 or more courses. In a word, results do improve with retreatment but so do the adverse events. A 2nd course of thalidomide treatment should be prescribed with great caution according to risk and benefit of the medication.

Double balloon enteroscopy (DBE) was 1st described in 2001 by Yamamoto et al.^[37]^ It allows deeper intubation of the small bowel (SB) compared with traditional endoscopes and endoscopic treatment of SB disorders. As a general statement, there has been a paucity of data regarding outcomes after endoscopic treatment of SB angioectasia. Samaha et al^[38]^ reported that 97% (129/133) patients with SB vascular lesions were successfully treated with argon plasma coagulation (using DBE). But rebleeding occurred in 45/98 (46%) patients at 36 months. Other recent studies also reported a high rebleeding rate about 35% to 45%.^[39,40]^ The high rebleeding rate may be related with the multiple and diffuse characteristic of SB vascular lesions. As to thalidomide treatment, there is few study concerned about its rebleeding rate. In our retrospective study, an overall response rate of 79.5% (62/78) and a relative lower recurrence rate of 21.0% after thalidomide treatment of GIVM were reported. Besides, in 2015, ACG Clinical Guideline^[41]^ also recommend that if a source of bleeding is found by VCE and/or deep enteroscopy in the small intestine that is associated with significant ongoing anemia or active bleeding, then the patient should be managed with endoscopic therapy (strong recommendation, low level of evidence). If bleeding persists or recurs or a lesion cannot be localized consideration may be given to medical treatment with iron, somatostatin analogs, or antiangiogenic therapy (strong recommendation, moderate level of evidence). So, we think endoscopic treatment under DBE may be much better for those isolated or limited vascular lesions, while thalidomide may be suitable for those multiple and diffuse lesions.

The major limitation of this study is that we could not carry out a prospective randomized controlled trial. The natural history of bleeding episodes in GI vascular malformations is unknown and can be very episodic in some patients. In this study, the effective rate of thalidomide treatment and retreatment for GIVM bleeding was defined based on patients’ by self-reporting. Concomitant therapies such as blood transfusions, iron supplementation, use of hemostatic drugs, and any other symptomatic treatments were performed as necessary during the whole study. They might mask some of the outcomes like increase in hemoglobin and change in stool color with iron. Self-reporting of stool color might introduce some bias. The strict exclusion criteria by which patients with severe comorbidities were excluded may influence the conclusions. Additional well-designed randomized trials are still required to confirm these findings.

In conclusion, thalidomide might be effective for patients with GIVM bleeding when they relapse or worsen after the 1st course of treatment, but side effects should be closely monitored.
